# Antitumor immunostimulatory activity of the traditional Chinese medicine polysaccharide on hepatocellular carcinoma

**DOI:** 10.3389/fimmu.2024.1369110

**Published:** 2024-02-22

**Authors:** Yang Liu, Jiawen Wu, Huiqin Hao

**Affiliations:** ^1^ College of Basic Medical Sciences, Shanxi University of Chinese Medicine, Jinzhong, China; ^2^ Basic Laboratory of Integrated Traditional Chinese and Western Medicine, Shanxi University of Chinese Medicine, Jinzhong, China

**Keywords:** hepatocellular carcinoma, traditional Chinese medicine, polysaccharides, antitumor immunostimulatory, Toll like receptor, mannose receptor

## Abstract

Hepatocellular carcinoma (HCC) is a prevalent malignancy, often associated with compromised immune function in affected patients. This can be attributed to the secretion of specific factors by liver cancer cells, which hinder the immune response and lead to a state of immune suppression. Polysaccharides derived from traditional Chinese medicine (TCM) are valuable constituents known for their immunomodulatory properties. This review aims to look into the immunomodulatory effects of TCM polysaccharides on HCC. The immunomodulatory effects of TCM polysaccharides are primarily manifested through the activation of effector T lymphocytes, dendritic cells, NK cells, and macrophages against hepatocellular carcinoma (HCC) both *in vivo* and *in vitro* settings. Furthermore, TCM polysaccharides have demonstrated remarkable adjuvant antitumor immunomodulatory effects on HCC in clinical settings. Therefore, the utilization of TCM polysaccharides holds promising potential for the development of novel therapeutic agents or adjuvants with advantageous immunomodulatory properties for HCC.

## 1 Introduction

According to the data provided by the World Health Organization (WHO), hepatocellular carcinoma (HCC) is the world’s sixth most common malignant tumor and the third leading cause of cancer death worldwide in 2020 (1). It is characterized by a short duration of illness and is difficult to heal, and the invasion, metastasis, and spread of HCC cells are the reasons for the high mortality rate of this disease (2). The conventional treatment of HCC is still mainly surgical resection, supplemented by drug therapy, but the disadvantage of surgical treatment of HCC is its high risk and challenging nature (3). The recurrence rate of surgical treatment is as high as 70% within 5 years (4). The effectiveness of traditional therapies for HCC is not satisfactory, so it is increasingly urgent to find new therapeutic methods for HCC. The early research on HCC mainly focuses on the tumor cells themselves. Still, with the deepening of the research, the focus has shifted to the surrounding tissue environment of the tumor. Especially, it has been reported that the occurrence, progression, and recurrence of HCC are closely related to its immune microenvironment (5–7).

The immune system has the functions of immune surveillance, defense, and regulation, and is composed of immune organs, immune cells, and immune active substances. The reason why tumors can escape the immune surveillance of the body is principally because they can produce excessive immunosuppressive factors, resulting in poor antitumor immune function in patients, which is not enough to produce a strong antitumor immune response to clear tumor cells (8–10). Therefore, the immunosuppressive microenvironment of HCC can provide a favorable condition for tumor growth, and immunotherapy is one of the effective methods to inhibit HCC growth (11).

Since the Japanese scholar Haoro Chihara first discovered in 1968 that *Lentinan* polysaccharide regulates tumor immune response, an upsurge in the research on the regulation of tumor immune response by Traditional Chinese Medicine (TCM) polysaccharides is booming (12). Polysaccharide is a linear or branched polymeric sugar composed of more than 10 monosaccharides connected by a glycosidic bond, which can be expressed by the general formula (C_6_H_10_O_5_) n (13). TCM polysaccharide is an effective polysaccharide extracted from a single Chinese medicine by modern scientific and technological methods. It mainly consists of glucose (Glu), rhamnose (Rha), mannose (Man), galactose (Gal), xylose (Xyl), arabinose (Ara), ribose (Rib), fructose (Fru), sucrose (Suc), fucose (Fuc), and other monosaccharide components (14, 15), which has anti-aging, anti-tumor, immune regulation and other functions (16–19). A large number of clinical trials and experimental studies have proved that some TCM polysaccharides (especially those extracted from the TCM with possible anti-tumor effect) have a certain function of regulating tumor immune response, with mild or no toxic and side effects at effective doses. For example, *Oenothera biennis* Polysaccharide (Evening primrose Polysaccharide) not only has a comparable antitumor effect to 5-Fluorouracil (5-FU) but has protective effects on liver and kidney function which is reflected in the low levels of alanine aminotransferase (ALT), aspartate aminotransferase (AST) and alkalines phosphatases (ALP), creatinine and urea in serum. Nonetheless, 5-FU administration to H22 tumor-bearing mice could not reverse the hepatic and renal damage, behaving a high level of ALT, AST, ALP, creatinine, and urea (20). However, TCM polysaccharides have large molecular weights and complex chemical structures, and the interaction mechanism between TCM polysaccharides and the immune system is still unclear.

In this article, the immunomodulatory effects of TCM polysaccharides on HCC, including their roles in regulating tumor adaptive immune response and innate immune response, were reviewed. We aim to provide a scientific basis for the development of novel therapeutic agents or adjuvants for this malignant tumor with beneficial immunomodulatory properties.

## 2 Role of TCM polysaccharides played in regulating tumor innate immune response

**Figure 1:**
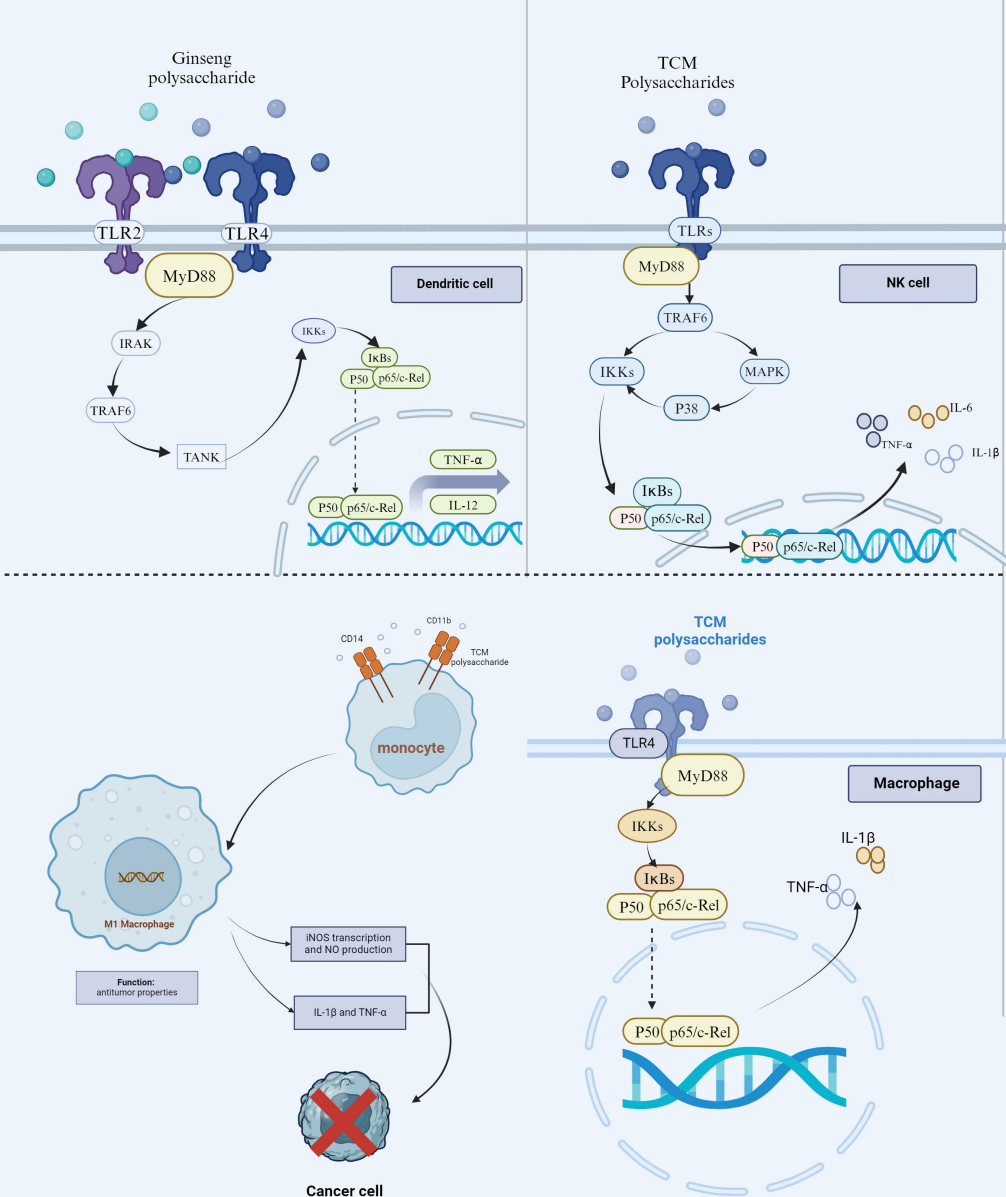
TCM polysaccharides’ role in regulating tumor innate immune response. TCM polysaccharides (such as *Ginseng* polysaccharides) can trigger the DCs to secrete the TNF-α and IL-12 through the typical NF-κB signaling pathway by binding to TLR4/TLR2. Meanwhile, TCM Polysaccharides promote NK cells to kill tumor cells by directly recognizing the TLRs-MyD88 complex and eventually activating the MAPK and NF- κ B signaling pathway to produce TNF-α, IL-1β, and IL-6. TCM Polysaccharides (such as *Astragalus membranaceus* Polysaccharide) enhance the differentiation of monocytes into M1 macrophages through the combination with CD11 and CD14/TLR-4 complex. The elevated transcription of iNOS and expression of NO, TNF-α, and IL-1β is helpful in inhibiting tumor growth. TCM Polysaccharides can also activate the macrophages directly by binding to the TLR4.

There are many types of cells of the myeloid lineage involved in innate immunity, including dendritic cells (DCs), natural killer (NK) cells, macrophages, monocytes, polymorphonuclear cells, mast cells, and so on. The roles of innate immunity in antitumor responses are related to that innate immune cells can detect tumors via some receptors, induce and amplify adaptive immune responses (such as that DCs are required for T-cell activity against tumors as the antigen-presenting cells), and exert direct effector responses (such as phagocytosis for macrophages and polymorphonuclear cells, and natural cytotoxicity for NK cells) (21). Toll-like receptors (TLRs) and some other pattern recognition receptors (PRRs) or integrin receptors are common receptors that play a crucial part in the innate immune system and may the critical targets for activating the antitumor response of the innate immune cells by TCM polysaccharide (**Figure 1**).

### 2.1 TCM polysaccharides involved in immune regulation of DCs

DCs are the initiators of the immune response (22). It has a strong antigen-phagocytosis in the immature, while a strong antigen-presenting function after maturity. DCs can stimulate the activation of initial T lymphocytes and play a key role in inducing anti-tumor immune response (23, 24). Studies have found that although there was sufficient DCs infiltration in the tumor microenvironment, these cells lose their due function of presenting tumor antigen and activating T lymphocytes, as well as are prone to apoptosis (25). Recently, studies have shown that promoting the maturation of DCs and enhancing their antigen-presenting effect can play a significant role in tumor treatment and vaccine preparation (26, 27).

TCM polysaccharides, such as *Ginseng* Polysaccharide (28), *Rehmannia* Polysaccharide (29), and *Laminaria* Polysaccharide (30), could also possess significant pharmacological effects against HCC. *Ginseng* Polysaccharide (31), *Rehmannia* Polysaccharide (32), and *Laminaria* Polysaccharide (33) can promote the maturation of bone marrow DCs (BMDCs), which were extracted from the femurs and tibias of female C57BL/6 mice, by down-regulating the activity of acid phosphoenzyme. After being treated with these TCM polysaccharides, the matured BMDCs manifested decreased phagocytosis activity and increased antigen-presenting ability. Furthermore, the decreased number of lysosomes, upregulated expression of membrane molecules at the protein level (including CD80, CD83, CD86, CD40, and MHC II), enriched secretion of interleukin (IL)-12 and tumor necrosis factor (TNF)-α could also be detected. All these pharmacologic effects of TCM polysaccharide together induced the T lymphocytes to be active to produce a powerful anti-tumor immune response at last.

Further empirical studies for the mechanism of activating the BMDCs by *Ginseng* polysaccharide have been proceeded *in vivo* with male Kunming mice and *in vitro* with BMDCs and T cells isolated from the BALB/c mice, because Ginseng is one of the most commonly used Chinese medicinal materials for anti-HCC (34). *Ginseng* Polysaccharide can promote the maturity of BMDCs by TCM Polysaccharide binding to the PRRs, such as TLR 4, TLR 2, and mannose receptor (MR) because Ginseng Polysaccharide consists of different monosaccharide components, including glucose, mannose fractions, galactose, and galacturonic acid fractions, etc. The glucose and mannose fractions are prone to be identified by TLR2 and MR, while galactose and galacturonic acid fractions can be recognized by TLR4 (35, 36). Once binding to TLR4/TLR2, the MyD88-mediated NF-κB signaling pathway could be triggered by *Ginseng* polysaccharide, leading to the functional maturation of BMDCs and the secretion of the proinflammatory cytokines (including TNF-α and IL-12). *Ginseng* Polysaccharide-induced secretion of IL-12 and TNF-α protein from BMDCs can be inhibited by admiration of antibodies against TLR2 and TLR4 (37). Therefore, the TLR4/TLR2-MyD88-NF-κB signaling pathway may play a vital role in the DCs-mediated anti-HCC effect induced by Ginseng Polysaccharide.

### 2.2 TCM polysaccharides involved in immune regulation of NK cells

NK cells are derived from bone marrow lymphoid stem cells, which do not express specific antigen-recognition receptors (38). They can recognize themselves and non-themselves through surface activating receptors and inhibitory receptors without antigen pre-stimulation, and directly kill tumor cells and virus-infected target cells by secreting granzyme and perforin (39, 40). Nowadays, NK cells have been considered an attractive, but underexplored, therapeutic target in HCC because of their role in immunosurveillance and their ability to eliminate malignant tumor cells (41). It was found that polysaccharides from various TCMs can improve the activity of NK cells to kill tumor cells, such as *Oenothera biennis* Polysaccharide (20), *Gynostemma pentaphyllum* Polysaccharide (42), and *Kaempferia galanga L* Polysaccharide (43). In some *in vivo* studies with H22 tumor-bearing mice, these three TCM Polysaccharides all could effectively inhibit the solid tumor growth of H22 hepatocarcinoma transplanted in mice, as well as elevate the body weight, spleen/thymus indexes and splenocyte proliferation of mice, through promoting the activity of NK cells in tumor-bearing mice.

Like some pathogen-associated molecular patterns (such as peptidoglycan and Lipopolysaccharide), TCM Polysaccharides can activate NK cells to kill tumor cells by directly recognizing TLRs, because they always have the components of glucose, galactose, and galacturonic acid fractions. Once these components bind to TLRs expressed on the NK cell’s surface, the TLRs-MyD88 complex will be formed to promote the activity of downstream transcription factors, including mitogen-activated protein kinases (MAPKs) and NF-κB, and the phosphorylation of its downstream proteins. It has been reported that the TCM Polysaccharides could enhance NK cell cytotoxicity against tumor cells via TLR4/MAPKs/NF-κB pathway, promote the TLR4-dependent production of interferon (IFN)-γ, and upregulate the protein level expression of CD69 in spleen NK cells (44, 45).

Taken together, these findings indicate that TCM Polysaccharides (at least including *Oenothera biennis* polysaccharide, *Gynostemma pentaphyllum* Polysaccharide, and *Kaempferia galanga L* Polysaccharide) have anti-HCC activity partly via improving the immune responses of NK cells, and the TLRs on the surface of NK cells may be the important binding-sites.

### 2.3 TCM polysaccharides involved in immune regulation of tissue-resident macrophages

The tissue-resident macrophages, like Kupffer cells in the liver, are characterized by their persistence in adults and associated with specialized tissue cells stably and closely (46). They have a variety of PRRs, modulatory receptors, and cytokine receptors on their surface, which can directly kill targets (such as pathogens, intracellular parasitic bacteria, and tumor cells), participate in the inflammatory response, process, and present antigens to initiate adaptive immune responses and secrete cytokines to regulate the immune response (47). In response to different stimuli and signaling, macrophages with antitumor properties or with a tumor-promoting capacity are classified as M1-like or M2-like phenotype (48). Tumor cells take advantage of this feature to generate more M2 cells and thus promote their growth (48). Zebrafish inflammation model studies have proved that the immunomodulatory effect of TCM polysaccharides may be related to the regulation of macrophage phagocytosis and the enhancement of antigen-presenting function (49, 50). For instance, *Platycodon grandiflorus* Polysaccharide was found to markedly activate iNOS transcription and NO production in RAW 264.7 cells and mouse peritoneal macrophages isolated from wild-type C3H/HeN, and anti-CD14 or anti-CD 11b antibody decreased NO production after the administration of *Platycodon grandiflorus* Polysaccharide in spleen-resident macrophages in BDF1 mice (51, 52). CD14, as an anchoring protein and a ubiquitous PRR, is primarily understood to act as a co-receptor for TLRs to activate innate immunity responses to pathogens and tissue injury (53). As the α-chain of integrin receptor CD11b/CD18 (also known as α_M_β_2_, Mac-1, and CR3), CD11b is highly expressed on the surface of innate immune cells, including macrophages and neutrophils. It is a new target for tumor immunotherapy. When CD11b activity is elevated, the number of M1 macrophages increases, while when CD11b becomes less, it leads to more M2 cell development (54). Therefore, it was indicated that CD14 and CD11b on the surface of macrophage may be the possible cellular binding sites of this TCM polysaccharide to exert anti-tumor effects. It has been proved that CD14 and CD 11b were the binding sites and effect targets of *Platycodon grandiflorus* Polysaccharide to macrophage, nevertheless, *Platycodon grandiflorus* Polysaccharide did not promote the proliferation of T cells, the expression of IL-2 in Th1 cells, or the expression of IL-4 in Th2 cells (52).

For another example, *Astragalus membranaceus* Polysaccharide could enhance the anti-tumor function of peritoneal macrophages isolated from BALB/c and C3H/HeJ mice by inducing them to produce cytokines (including TNF-α and IL-1β), and this effect can be eliminated in TLR-4 mutant C3H/HeJ mice or through the injection of TLR4 antibodies, proving that promotion the secretion of cytokines in macrophages by *Astragalus membranaceus* Polysaccharide is possibly related to the TLR4 binding (55).

When it comes to treating hepatocellular carcinoma, *Astragalus membranaceus* Polysaccharide could enhance the expression of M1 macrophage biomarkers (such as iNOS, IL-1β, and TNF-α) and M1 macrophage proportions, as well as could reduce the expression of M2 macrophage biomarkers (including IL-10, Arg-1) and M2 macrophage proportions in the tumor-associated macrophages (TAMs)/MHCC97H cells and TAMs/Huh7 cells co-cultured models. Moreover, the *Astragalus membranaceus* Polysaccharide-mediated elevation of the M1 phenotype of TAMs could significantly repress the ability of proliferation, migration, and invasion both in MHCC97H and Huh7 cells. On the other side of the coin, *Astragalus membranaceus* Polysaccharide could also enhance the M1/M2 macrophage ratio in the tumor tissues of Huh7-bearing BALB/c nude mice model and thus inhibit the growth of HCC *in vivo* (56). This anti-HCC effect of *Astragalus membranaceus* Polysaccharide may have a close relationship with the combination of CD11 and CD14/TLR-4 complex.

So to summarize, regulating the activity of innate immune cells, including DCs, NK cells, and macrophages through the membrane receptors such as TLRs, is one of the important mechanisms by which TCM polysaccharides exert the anti-HCC effect.

## 3 Role of TCM polysaccharides played in regulating tumor adaptive immune response

**Figure 2:**
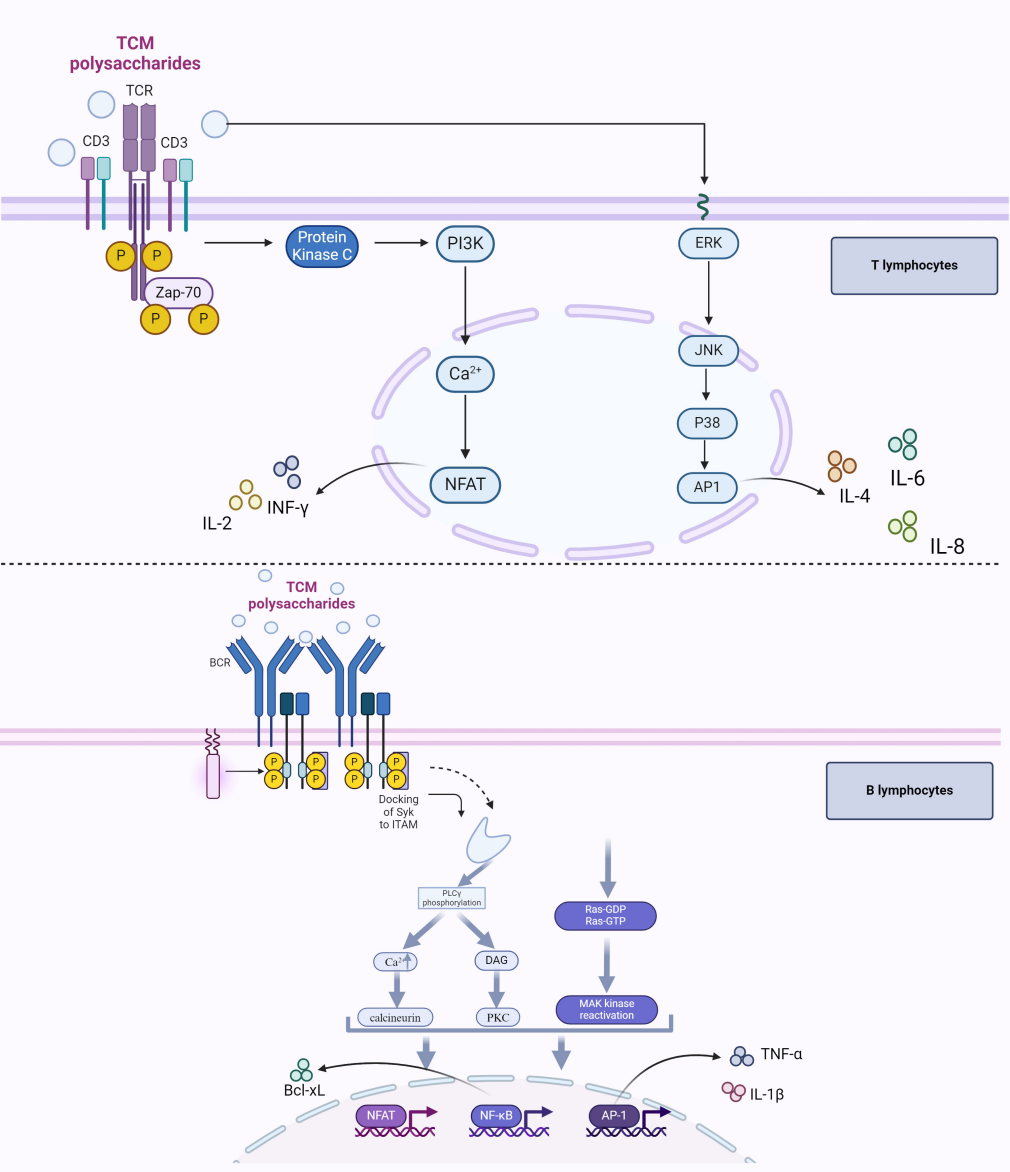
TCM polysaccharides’ role in regulating tumor adaptive immune response. Once recognized directly or indirectly by the TCR, TCM polysaccharides activate the PTK, and the latter plays an important role in antitumor by regulating the expression of cytokines, including IL-2, IL-4, IL-6, IL-8, and INF-γ, via PI3K or MAPK signaling pathway. B lymphocytes can also recognize TCM polysaccharides through mIg/CD79 complex or TLR 2/4 signaling cascades, which will lead to the elevation of nuclear translocation of NFAT/AP-1/NF-κB and regulate the expression of B lymphocyte-related genes, through the activation of the calcineurin/PKC/MAPK (ERK) signaling cascade.

T lymphocytes and B lymphocytes are the main effector cells in the anti-tumor adaptive immune response (57, 58). However, the tumor itself can produce many immunosuppressive factors to repress the activation of effector T lymphocytes (Teffs) and B lymphocytes, thus, the immune microenvironment of the patients with tumors is in a state of immunosuppression (59, 60). Many TCM polysaccharides have strong activation of Teffs and B lymphocytes and can inhibit the growth of tumor cells (61). The molecular pathway of T lymphocytes activated by TCM polysaccharides is mainly through the T-cell receptor (TCR)/CD3 complex-mediated signaling pathway (including MAPK signaling pathway), while the regulatory effect on B lymphocytes activated by TCM polysaccharides is mainly bound to the IgM/CD79 complex receptor or TLR2/4 on the cell surface, and the MAPK and NF-κB signal transduction pathways are also involved in B lymphocytes activation (**Figure 2**).

### 3.1 Direct and indirect recognition of TCM polysaccharides by T lymphocytes

It is well known that many polysaccharides can be treated by MHC Class II molecules and presented to T cells for indirect recognition by TCR. TCM Polysaccharides could be degraded into small molecular weight carbohydrates by the NO system via the endocytosis pathway, and these small molecular weight carbohydrates can be processed and presented by MHC Class II molecules and recognized by TCR molecules (62). Brian (63) has demonstrated by laser confocal method that the adhesion of antigen-presenting cells and T lymphocytes occurred after the addition of polysaccharide antigens, and the co-localization of MHC Class II molecules, TCR molecules, and polysaccharide antigens could also be detected simultaneously. Moreover, polysaccharides can also be directly recognized by T lymphocytes in two ways. One is that polysaccharides can be presented to γδ T cells by CD1 complex via intracellular pathways (64), and another is that polysaccharides (as a kind of hapten) can be simultaneously presented by MHC class II molecules and recognized by TCR molecules, in the form of glycoprotein or sugar linkage (65).

Once recognized directly or indirectly by the TCR, TCM polysaccharides will possess good immune-promoting effects by activating the protein tyrosine kinase (PTK) (66). PTK will upregulate the expression of genes involved in immune response (including IL-2, IL-4, IL-6, IL-8, and INF-γ) at both the transcriptional and translational levels through the PI3K or MAPK signaling pathway: (1) The protein kinase C (PKC) can be stimulated to migrate from cytoplasm to membrane by PTK via PI3K signaling pathway and result in the increase of Ca^2+^ concentration in cytoplasm. After being activated by the intracytoplasmic accumulation of Ca^2+^, calcineurin induces the nuclear translocation of the nuclear factor of activated T cells (NAFT) and elevates its transcription factor activity, leading to a high level of expression of IL-2 and INF-γ. (2) PTK can induce the nuclear translocation of activator protein 1 (AP1) via the MAPK signaling pathway (ERK/JNK/p38). As a transcription factor associated with immune regulation, AP1 in the nucleus can induce gene expression, such as IL-4, IL-6, and IL-8 (67, 68).

### 3.2 Recognition of TCM polysaccharides by B lymphocytes

#### 3.2.1 Membrane immunoglobulin complex receptor-mediated recognition

The membrane immunoglobulin (mIg) receptor is the most important receptor and characteristic sign on the surface of B lymphocytes. It can form a mIg complex receptor with CD79b, which is known as the B cell receptor (BCR), to recognize antigens and regulate B lymphocyte activation. When mIg/CD79 binds to TCM polysaccharides, the calcineurin/PKC/MAPK (ERK) signaling cascade will be activated to induce the nuclear translocation of NFAT/AP-1/NF-κB, which will regulate the expression of B lymphocyte-related genes (69).

#### 3.2.2 TLR 2/4 signaling cascades mediated recognition

TLR2/4 receptor also exists on the surface of B lymphocytes, and TCM polysaccharide can bind to the TLR2/4 receptor on the surface of B lymphocytes to regulate the transcription of related genes via the MAPK signaling pathway or NF-κB signaling pathway. Studies have shown that *Acanthopanax* Polysaccharide can significantly stimulate the proliferation of B lymphocytes in C3H/HeN mice by activating MAPKs (such as Erk1/2, p38, and JNK), and the transcription factor NF-B, but when cultured with TLR4 antibody or TLR2 antibody, the proliferation of B lymphocytes is significantly inhibited. These results indicated that Acanthopanax polysaccharide could bind to the TLR2/4 receptor on the surface of B lymphocytes to promote the proliferation of B lymphocytes (70). However, polymyxin B (PMB), a specific inhibitor of LPS, did not significantly affect the activities of Acanthopanax polysaccharide on B lymphocytes, which indicated that Acanthopanax polysaccharide could activate the TLR signaling cascades differently from LPS.

To summarize briefly, activating the T lymphocytes and B lymphocytes is another important mechanism for TCM polysaccharides to anti-HCC. The TCR, BCR, and TLRs are the main binding sites for TCM polysaccharides to induce the activation of immune cells in the adaptive immunity system.

## 4 Experimental and Clinical trials on HCC of TCM polysaccharides by modulating immune response

### 4.1 Experimental trial on HCC of TCM polysaccharides by modulating immune response

#### 4.1.1 *Trametes robiniophila Murr* polysaccharide

*Trametes robiniophila Murr* is a kind of light brown agaric growing on the trunk of the Sophora tree, belonging to *Agaricales* and *Basidiomycetes*. It is also known as Huaier and has been used in traditional Chinese medicine for nearly 1,600 years and was first documented by Li Shizhen of the Ming Dynasty (71). In recent decades, *Trametes robiniophila Murr* has been discovered and used as a supplement in the treatment of cancer, especially has achieved satisfactory results for anti-HCC (72, 73). The accumulated evidence shows that the anti-tumor mechanism of *Trametes robiniophila Murr* is mainly to inhibit the proliferation of endothelial cells, interfere with the formation of tumor blood vessels, activate the immune system, induce the apoptosis of tumor cells, and inhibit the proliferation of tumor cells (74, 75). *Trametes robiniophila Murr* Polysaccharide, one homogenous polysaccharide purified from Huaier, exerted more potent antitumor activity in inhibiting HCC growth (76). In an HCC H22-based mice experiment, *Trametes robiniophila Murr* Polysaccharide significantly repressed the growth and the pulmonary metastasis of the transplanted H22 solid HCC *in vivo*. While prolonging the live time of mice bearing H22 tumors, *Trametes robiniophila Murr* Polysaccharide elevated the relative weight of immune organs (spleen and thymus) and lymphocyte proliferation reaction notably, including an increasing percentage of CD4^+^ T cells and natural killer (NK) cells, whereas a decreasing number of CD8^+^ T cells. Besides, the administration of *Trametes robiniophila Murr* Polysaccharide promoted the secretion of immune-stimulating serum cytokines (IL-2 and IFN-γ) but inhibited the production of immune-suppressing serum cytokines (IL-10) in H22-bearing mice (77). It suggested that *Trametes robiniophila Murr* Polysaccharide could exhibit prominent antitumor activities via enhancement of host immune system function, and could be developed individually as a potent immunological response modifier for HCC therapy.

#### 4.1.2 *Salvia miltiorrhiza* polysaccharide

*Salvia miltiorrhiza* (also named Danshen) is the dried root of the Chinese medical plant Salvia miltiorrhiza Bunge (Labiatae), and in the clinical practice of TCM, it has long been used for clearing heat for detumescence, soothing the nerves and tranquilizing the mind (78, 79). In line with the Chinese medicine theory, *Salvia miltiorrhiza* was commonly used for the prevention and treatment of myocardial infarction, apoplexy, hepatitis, tumors, and immunological disorders (80, 81). At the same time, *Salvia miltiorrhiza* has shown a good application prospect for the treatment of liver fibrosis and HCC (82–84). Polysaccharide is one of the main immunomodulatory active components in the water extract of *Salvia miltiorrhiza*, and it can dose-dependently promote the proliferation of T lymphocytes and B lymphocytes of the cancer patients and significantly improve the cytotoxicity of T lymphocytes against cancer cells, as well as up-regulated the gene expression of cytokines including IL-4, IL-6, and IFN-γ (85, 86). It has been proved that *Salvia miltiorrhiza* Polysaccharide can suppress the proliferation of H22 cells on tumor-bearing mice *in vivo*, by alleviating the apoptosis of tumor transplantation-induced CD4^+^ T cell and dysregulating of serum cytokine profiles (such as prostaglandin E2), as well as elevating the cytotoxic activities of NK cells and CD8^+^ T cells (87). Moreover, *Salvia miltiorrhiza* Polysaccharide also substantially declined the intracellular cyclic adenosine monophosphate (cAMP) in CD4^+^ T cells, which subsequently elevated the protein levels of JAK3 and enhanced the STAT5 or STAT5 phosphorylation-dependent expression of anti-apoptotic genes (87). These works provided a new perspective for the application of *Salvia miltiorrhiza* against HCC and offered experimental evidence for using *Salvia miltiorrhiza* Polysaccharide as an adjunct reagent in the clinical treatment of HCC.

#### 4.1.3 *Ganoderma lucidum* polysaccharide

As a versatile traditional Chinese herb and nutraceutical, *Ganoderma lucidum* has been applied to treat multiple diseases in the clinical practice of TCM, including digestive disorders, cardiovascular system disease, and nervous system disease (88–90). Among all of its extracts, *Ganoderma lucidum* Polysaccharide is the main bioactive component and possesses many therapeutic effects, such as antitumor, immunoregulatory, anti-oxidant, and anti-neurodegenerative activities (91–93). It has been reported that *Ganoderma lucidum* Polysaccharide is capable of suppressing the growth of tumors in hepatoma-bearing Kunming and BALB/c mice associated with an increase of the ratio of Teffs to Tregs, through increasing the secretion of IL-2 to eliminate the suppression effect of Treg on the proliferation of Teffs (94). The ability of *Ganoderma lucidum* Polysaccharide to suppress the proliferation of Treg is possibly related to inhibiting the gene transcription level expression of Foxp3 by elevating the expression of miR-125b, and in the hepatoma-bearing mice, miR-125b inhibitor abolished the effect of Ganoderma lucidum Polysaccharide on tumor growth (94).

#### 4.1.4 *Strongylocentrotus nudus* eggs polysaccharide

*Strongylocentrotus nudus* is another kind of precious traditional Chinese medicine, which has the remarkable effect of anti-tumor (95). As a D-glucan containing an α-1, 4-linked backbone, and α-1, 3-linked branches, *Strongylocentrotus nudus* Egg Polysaccharide is extracted and purified from sea urchins’ eggs (96). *Strongylocentrotus nudus* Egg Polysaccharide is widely accepted as a bioactive anticancer compound with profound inhibitory effects on tumor growth closely related to its immunomodulatory biological activity (44, 97). Numerous pharmacological studies have indicated that *Strongylocentrotus nudus* egg polysaccharide is an anticancer candidate working by activating T lymphocytes and B lymphocytes (98, 99). In several experiments of a mouse model of H22 hepatocellular carcinoma, *Strongylocentrotus nudus* Egg Polysaccharide could inhibit the tumor growth of H22-bearing imprinting control region (ICR) mice and stimulate the proliferation of T lymphocytes and B lymphocytes in a dose-dependent manner, involving not only remarkably enhance the spleen and thymus indices, CD4^+^ and CD8^+^ T cell numbers as well as CTL activity, but it also elevated IgA, IgM and IgG levels in the serum. At the same time, the IL-2 and TNF-α secretion in serum also elevated after the admiration of *Strongylocentrotus nudus* Egg Polysaccharide (98, 100). Furthermore, *Strongylocentrotus nudus* Egg Polysaccharide could also induce the proliferation of splenocytes isolated from the ICR mice *in vitro*, and this effect could be inhibited by the TLR2 and TLR4 monoclonal antibodies, which indicated that *Strongylocentrotus nudus* Egg Polysaccharide may mediate splenocyte proliferation via TLR2 and TLR4 (100). These studies demonstrate that *Strongylocentrotus nudus* Egg Polysaccharide can effectively inhibit HCC via enhancement of adaptive immune system function, and it could be a potential therapeutic drug for hepatocarcinoma.

#### 4.1.5 *Radix Glycyrrhizae* polysaccharide

Treg cells in the peripheral blood of tumor patients (including HCC) increased significantly and inhibited the function of dendritic cells (DCs) and Teffs through the secretion of IL-10 and transforming growth factor (TGF)-β, thus inhibiting the anti-tumor immune response of the body (101, 102). Increased levels of Treg cells have been detected in the peripheral blood, the primary tumor microenvironment, and the draining lymph nodes of cancer patients (103–105). Therefore, interventions targeting Treg cells have become the new strategy of many therapies for malignant tumors (106, 107). *Radix Glycyrrhizae* is a commonly used traditional Chinese herbal medicine with a wide range of pharmacological functions throughout Chinese history, and the polysaccharide is a major active compound extracted from it, which is cytotoxic to cancer cells (108, 109). An *in vivo* experimental study with H22 hepatocarcinoma-bearing BALB/c mice has shown that *Radix Glycyrrhizae* Polysaccharide could down-regulate the population of Treg cells and decrease the expression of Foxp3 mRNA and IL-10 mRNA in the lymph node. At the same time, *Radix Glycyrrhizae* Polysaccharide treatment could also influence the level of the Th1/Th2 cytokines in serum by decreasing the level of IL-10 and TGF-β and increasing the level of IL-2 and IL-12p70 (110). It was indicated that the mechanism of *Radix Glycyrrhizae* Polysaccharide on lowering the proportion of Treg cells may be related to the regulation of the expression of Foxp3 and balance of the secretion of Th1/Th2 cytokines.

### 4.2 Clinical trials on HCC of TCM polysaccharides by modulating immune response

The traditional clinical treatment of HCC is surgery, radiotherapy, and chemoradiotherapy. At present, immunotherapy has become one of the most promising areas in tumor therapy, and regulating the biological function of immune cells has been proven to be a powerful weapon to exert anti-HCC effects, and has been applied more and more in clinical practice (111, 112). As natural active macromolecules with a wide range of sources, TCM polysaccharides can achieve anti-tumor effects by enhancing immune regulation, inhibiting tumor growth, tumor cell invasion and metastasis, and the development of tumor microenvironment (TME). Compared with the traditional anti-tumor treatments, TCM polysaccharides show the advantages and characteristics of multi-pathway and multi-target action. At the same time, according to some clinical observational studies or randomized controlled trials (RCT), it can improve the sensitivity of tumor cells to radiotherapy and chemotherapy, reduce adverse reactions, reduce the risk of tumor resistance and recurrence, and play a positive role in HCC treatment (113). Therefore, the participation of TCM polysaccharides in the adjuvant treatment during or after tumor surgery or chemoradiotherapy has attracted more and more attention from researchers, and many TCM polysaccharides have been used in clinical adjuvant therapy on HCC to enhance clinical efficacy and reduce toxic side effects.

The most widely used TCM polysaccharide in the clinical treatment of HCC is *Lentinan* polysaccharide. The results of three RCT researches involving 374 HCC patients, which were conducted in Shandong Province, Henan Province, and Tianjin City in China, showed that compared with the patients that only treated with transcatheter arterial chemoembolization (TACE), administration of Lentinan polysaccharide after TACE could increase the tumor volume reduction rate, NK cell activity, CD4^+^ T cell level, CD4^+^/CD8^+^ ratio and the content of IL-2 in serum, as well as decrease the level of CD8^+^ T cells and the content soluble IL-2 receptor (sIL-2R) in serum. However, there was no statistical difference in alpha-fetoprotein (AFP) reduction rate between the two treatments (114, 115). Another meta-analysis involving 7 randomized controlled trials, a total of 577 HCC patients, showed that compared with TACE alone, *Lentinan* polysaccharide adjuvant TACE may improve the 1-year survival rate of HCC patients, and increase the number of CD4^+^ T cells and CD4^+^/CD8^+^ ratio in peripheral blood of patients (116). Moreover, intraperitoneal injection of *Lentinan* polysaccharide in the treatment of advanced HCC patients with ascites could also improve the symptom remission rate and Karnofsky Performance Status (KPS) score (117). All the results indicated that *Lentinan* polysaccharide has certain advantages in the therapy of HCC as an adjuvant by improving the immunity of the patients. In addition to positively regulating the activation of Teffs, *Lentinan* polysaccharide can also negatively regulate the proliferation of Treg cells in HCC patients. In a clinical observational trial involving 46 HCC patients, *Lentinan* polysaccharide could downregulate the proportion of Treg cells in the peripheral blood of the patients, and it would be more effective when administered immediately after TACE operation (118). These results suggested that *Lentinan* polysaccharide could significantly improve the immune status and promote the reconstruction of immune function of HCC patients after TACE operation, by reducing the toxic side effects of TACE therapy, inhibiting the growth of hepatocellular carcinoma cells and promoting the recognition of hepatocellular tumor antigen.

Overall, although the efficacy and the mechanism still need to be further evaluated and revealed, TCM polysaccharides do have very attractive potential in anti-HCC, according to the current studies. It is necessary to further improve the experimental design of laboratory studies and clinical trials, to evaluate the application prospect of TCM polysaccharides in the treatment of HCC more objectively.

## 5 Deficiency and prospects

The detailed information on the TCM polysaccharides involved in this review is exhibited in **Table 1** (20, 119–129), and their application in treating HCC in animal experiments and clinical trials has been summarized in **Table 2**. And in **Table 3**, the mechanism of action of different TCM polysaccharides with different immune cells was also exhibited. Based on the current research status in this field, TCM Polysaccharide, as an adjunct therapeutic drug, may have a very broad application prospect in the clinical treatment of HCC. It is intriguing to speculate that TCM polysaccharides can directly activate innate immune response or adaptive immune response to anti-HCC by mediating TLR signaling because natural TCM has been safely used for a long period in many oriental countries. Moreover, they have not been associated with any detrimental tissue injuries, which can be caused by LPS, at their biologically effective concentrations.

**Table 1:** The detailed information on the TCM polysaccharide in this review.

Polysaccharide	Molecular weight	Monosaccharide composition	Species classification of herbs	Preparation methods
*Trametes robiniophila Murr* Polysaccharide (119)	30 KDa	Glu, Man, Rha, Gal, Xyl, Rib, Ara,	*Dioscoreaceae*	DEAE fiber column layers Analysis method; Dextran gel column chromatography method
*Salvia miltiorrhiza* Polysaccharide (120)	43-569 KDa	Glu, Gal, Ara, Rha, GalUA, Galin, Glc,	*Lamiaceae*	Water extraction and alcohol sedimentation method; hot water immersion method; Ultrasonic extraction
*Ganoderma lucidum* Polysaccharide (121)	22 KDa	Man, Glu, Gal	*Polyporaceae*	Boiling water reflux method
*Strongylocentrotus nudus* eggs Polysaccharide (122)	678 KDa	Glu	*Strongylocentrotus nudus*	Water extraction with reduced pressure concentration
*Lentinan* Polysaccharide (123)	400-800 KDa	Xyl, Man, Ara, Gal, Rha, Glu	*Umbelliferae*	Water extraction and alcohol precipitation
*Radix Glycyrrhizae* Polysaccharide (124)	10-13 KDa	Glc, Gal, Ara, Rha, Man	*Leguminosae*	Ultrasonic extraction
*Ginseng* Polysaccharide (125)	3.5-110 KDa	Ara, Rha, Fuc, Xyl, Man, Gal, Glu	*Araliaceae*	Wet crushing and extraction method
*Rehmannia* Polysaccharide (126)	11-43 KDa	Glu, Gal, Man, Rha, Xyl, Ara, Fuc	*Scrophulariaceae*	Water extraction and alcohol precipitation
*Laminaria* Polysaccharide (127)	5-27 K Da	Ara, Man, Glu	Laminariaceae	Ultrasonic extraction
*Platycodon grandifloru*s Polysaccharide (128)	2-267 KDa	Man, Rha, Glc, Gal, Xyl, Ara	*Campanulaceae*	Hot water extraction; Ultrasonic-assisted extraction; Microwave-assisted extraction
*Astragalus membranaceus* Polysaccharide (129)	3.2-257.7 KDa	Gal, Glu, Rha, Ara	*Leguminosae*	Water extraction with ethanol precipitation
*Oenothera biennis* Polysaccharide (20)	9.8 Da	Glc, Gal, Man, Ara, Rha	*Onagraceae*	Ultrasonic-assisted extraction; High pressure extraction method

**Table 2:** The application of TCM Polysaccharide in treating HCC in animal experiments and clinical trials.

Polysaccharide	Purified or extracted from	Roles played in regulating HCC immune response	Animal experiments or clinical trials	References
*Trametes robiniophila Murr* Polysaccharide	*Trametes robiniophila Murr* (Huaier)	CD4^+^ T cells and NK cells ↑ serum IL-2 and IFN-γ ↑ CD8^+^ T cells ↓ serum IL-10 ↓	HCC H22- bearing mice experiment *in vivo*	(76, 77)
*Salvia miltiorrhiza Polysaccharide*	*Salvia miltiorrhiza* (Danshen)	cytotoxic activities of NK cells and CD8^+^ T cells ↑ CD4^+^ T cell apoptosis ↓ serum prostaglandin E2 ↓ cAMP in CD4^+^ T cell ↓ expression of JAK3 and STAT5 ↑	HCC H22-bearing mice experiment *in vivo*	(85–87)
*Ganoderma lucidum* Polysaccharide	*Ganoderma lucidum*	the ratio of Teffs to Tregs ↑ secretion of IL-2 ↑ expression of miR-125b ↑ expression of Foxp3 ↓	HCC H22-bearing mice experiment *in vivo*	(94)
*Strongylocentrotus nudus* eggs Polysaccharide	*Strongylocentrotus nudus*	spleen and thymus indices ↑ CD4^+^ and CD8^+^ T cell numbers ↑ CTL activity ↑ serum IgA, IgM, IgG, IL-2, TNF-α ↑	HCC H22-bearing mice experiment *in vivo* Splenocyte proliferation *in vitro*	(98–100)
*Radix Glycyrrhizae* Polysaccharide	*Radix Glycyrrhizae*	number of Treg cells ↓ mRNA expression of Foxp3 and IL-10 ↓ serum IL-10 and TGF-β ↓ serum IL-2 and IL-12p70 ↑	HCC H22-bearing mice experiment *in vivo*	(110)
*Lentinan* Polysaccharide	*Lentinus edodes*	number of Treg cells ↓ expression of Foxp3 and STAT3 ↓ CD4^+^ T cell ↑ CD4^+^/CD8^+^ ratio ↑ serum IL-2 ↑ CD8^+^ T cells serum sIL-2R ↓	Clinical observational studies or RCT with HCC patients	(114–118)

Red arrows represent increased expression levels of these indicators, while green arrows represent decreased expression levels.

**Table 3:** The mechanism of different TCM polysaccharides with different immune cells.

Polysaccharide	Immune cells	Binding targets	Signaling Pathway	References
*Ginseng* Polysaccharide	DCs	TLRs and MR	TLRs-MyD88 complex mediated NF-κB signaling pathway	(28–37)
*Rehmannia* Polysaccharide				
*Laminaria* Polysaccharide				
*Oenothera biennis* Polysaccharide	NK cells	TLRs	TLRs-MyD88 complex mediated MAPKs and NF-κB signaling pathway; TLR4-dependent IFN-γ and CD69 production	(20, 42–45)
*Gynostemma pentaphyllum* Polysaccharide				
*Kaempferia galanga L Polysaccharide*				
*Platycodon grandiflorus* Polysaccharide	tissue-resident macrophages	CD14/CD11b-TLR4 complex	Modulating the M1 macrophage polarization to elevate the transcription of iNOS, IL-1β, and TNF-α	(51, 52, 55, 56)
*Astragalus membranaceus* Polysaccharide				
*Trametes robiniophila Murr* Polysaccharide	T lymphocytes	TCR/CD3 complex	PTK-mediated PI3K/NAFT or MAPK signaling pathway	(66, 77, 87, 92)
*Salvia miltiorrhiza* Polysaccharide				
*Acanthopanax* Polysaccharide	B lymphocytes	mIg/BCR(CD79) complex or TLR2/4 receptor	mIg/BCR(CD79) complex-mediated calcineurin/PKC/ERK signaling pathway or LR2/4 receptor-mediated MAPK signaling pathway	(69, 70, 94, 100)
*Ganoderma lucidum* Polysaccharide				
*Strongylocentrotus nudus* egg polysaccharide				

However, there are still shortcomings in the research, and the clinical application prospects can be further improved from the following aspects: (1) Most of the current reports on the regulation of anti-tumor immunity by TCM polysaccharides focus on the effects on the number, phenotype, activity and signaling pathway of single immune cells, but ignore that the interaction of multiple cells in the tumor immune microenvironment also play a synergistic role in anti-tumor (130). The complexity and diversity of TCM polysaccharide structure make it have the characteristics of multi-pathway and multi-target action, which is particularly important for the prevention and treatment of systemic diseases, especially cancer, through the overall regulation of immune cells in the immune system (131). Therefore, it is one of the directions to break through the current research bottleneck to carry out research work from micro to macro, from single cell to cell network. (2) As bioactive macromolecules, TCM polysaccharides need to bind to pattern-recognition receptors on the cell membrane of immune cells to initiate immune regulation and cellular immune response. Different immune cells have different receptors, and polysaccharides from different TCM sources can bind to different receptors (35, 54, 132). Which receptor on the surface of immune cells is involved in TCM polysaccharide recognition and binding, as well as the induced cell cascade reaction has been a hot topic of research. However, the current research mainly focuses on the changes in signal molecules after polysaccharide action. Further studies on the types and functions of polysaccharide receptors can be used as another research direction to reveal the mechanism of specific targeting of TCM polysaccharides against immune cells to regulate and control the growth of HCC. (3) Intestinal microbiota is highly associated with the occurrence and development of HCC (133). The characteristic microbiota of the intestinal tract is significantly correlated with the function of immune cells in the host tumor immune microenvironment, and the dysbiosis of gut microbiota could increase bacterial translocation, which suppresses the activation of antigen-presenting cells such as DCs and macrophages and then leading to the declined IFN-γ/TNF-α production from T cell subsets to kill the tumor cells (134). Therefore, Intestinal microbiota can regulate the immune microenvironment of tumors and affect the development of tumors. TCM Polysaccharides can regulate the tumor immune microenvironment and play an anti-tumor role as prebiotics to maintain the diversity of intestinal microorganisms in the intestinal tract by proliferating the growth of beneficial bacteria and inhibiting the growth of pathogenic bacteria, thus activating the immune cells such as intestinal DCs and inducing the expansion of germinal centers (GC) to trigger the differentiation of the B cells (135). Moreover, the anti-tumor effects of TCM Polysaccharides probably also have a close relationship with the fecal short-chain fatty acid (SCFA) (136). SCFAs, including acetate, butyrate, and propionate, are the key productions from indigestible polysaccharides by bacterial fermentation (137). SCFAs can reduce the incidence of tumors by acting on B cells to secrete antibodies and increasing the level of aldehyde dehydrogenase in DCs to convert vitamin A to retinoic acid to activate the plasma cells (138, 139). Therefore, the research on “TCM polysaccharides-intestinal microbiota-tumor immune microenvironment-immune cells” can better explore the anti-tumor mechanism of TCM polysaccharides. (4) The biological activity of TCM polysaccharides is closely related to its primary and advanced structure, but the study of its structure-activity relationship is still a shortcoming in this field (140). Therefore, it is of great significance to further break through the key technical barriers of TCM polysaccharide preparation and characterization for the innovation and transformation of TCM polysaccharides and anti-tumor precision medicine. (5) We can also focus on the combined application of TCM polysaccharides with chemotherapy or immunotherapy drugs, the use of TCM polysaccharides as drug delivery carriers, and the construction of tumor-targeting ligands to regulate the tumor immune microenvironment and other research directions. (6) Clinical studies on the treatment of HCC by TCM polysaccharides are mainly carried out in China at present, and the research subjects are all Chinese patients (114–118), which may affect the conclusion of this study. At the same time, none of the literature mentioned the distribution hiding, blind method, loss of access, and intentionality analysis. It is suggested that a multi-center randomized controlled double-blind trial with a large sample should be carried out with a reasonable design and strict implementation to further verify the efficacy of TCM polysaccharides in treating HCC. In terms of methodology, full randomization and implementation of assignment concealment should be used to report trials according to consolidated standards of reporting trials (CONSORT) criteria, while long-term follow-up should be conducted including endpoint indicators (such as survival rate and median survival) and negative results reporting, to draw more reliable conclusions for clinical guidance.

## 6 Conclusion

TCM polysaccharides have a very broad application prospect in the research field of anti-HCC by regulating the innate and adaptive immune response. It is a key issue to accelerate and clarify the mechanism of TCM polysaccharides in regulating the immune regulation of HCC, which can provide a more comprehensive theoretical basis for the clinical application of TCM polysaccharides in tumor therapy and has important academic value and practical significance.

## Author contributions

YL: Writing – original draft, Writing – review & editing. JW: Writing – original draft. HH: Writing – review & editing.
